# Prognostic impact of corticosteroid and tocilizumab use following chimeric antigen receptor T-cell therapy for multiple myeloma

**DOI:** 10.1038/s41408-024-01048-0

**Published:** 2024-05-27

**Authors:** Bruno Almeida Costa, Jessica Flynn, Noriko Nishimura, Sean M. Devlin, Tasmin Farzana, Sridevi Rajeeve, David J. Chung, Heather J. Landau, Oscar B. Lahoud, Michael Scordo, Gunjan L. Shah, Hani Hassoun, Kylee Maclachlan, Malin Hultcrantz, Neha Korde, Alexander M. Lesokhin, Urvi A. Shah, Carlyn R. Tan, Sergio A. Giralt, Saad Z. Usmani, Karthik Nath, Sham Mailankody

**Affiliations:** 1 Department of Medicine, Cellular Therapy Service, Memorial Sloan Kettering Cancer Center, New York, NY USA; 2 Department of Medicine, Mount Sinai Morningside and West, Icahn School of Medicine at Mount Sinai, New York, NY USA; 3 Department of Epidemiology and Biostatistics, Memorial Sloan Kettering Cancer Center, New York, NY USA; 4 Department of Medicine, Myeloma Service, Memorial Sloan Kettering Cancer Center, New York, NY USA; 5 Department of Medicine, Adult Bone Marrow Transplantation Service, Memorial Sloan Kettering Cancer Center, New York, NY USA; 6 Department of Medicine, Weill Cornell Medicine, New York, NY USA

**Keywords:** Disease-free survival, Myeloma, Myeloma, Immunotherapy

## Abstract

Despite being the mainstay of management for cytokine release syndrome (CRS) and immune effector cell-associated neurotoxicity syndrome (ICANS), there is limited data regarding the impact of tocilizumab (TCZ) and corticosteroids (CCS) on chimeric antigen receptor (CAR) T-cell efficacy in multiple myeloma (MM). The present study aims to evaluate the prognostic impact of these immunosuppressants in recipients of BCMA- or GPRC5D-directed CAR T cells for relapsed/refractory MM. Our retrospective cohort involved patients treated with commercial or investigational autologous CAR T-cell products at a single institution from March 2017–March 2023. The primary endpoint was progression-free survival (PFS). Secondary endpoints included overall response rate (ORR), complete response rate (CRR), and overall survival (OS). In total, 101 patients (91% treated with anti-BCMA CAR T cells and 9% treated with anti-GPRC5D CAR T cells) were analyzed. Within 30 days post-infusion, 34% received CCS and 49% received TCZ for CRS/ICANS management. At a median follow-up of 27.4 months, no significant difference in PFS was observed between CCS and non-CCS groups (log-rank *p* = 0.35) or between TCZ and non-TCZ groups (log-rank *p* = 0.69). ORR, CRR, and OS were also comparable between evaluated groups. In our multivariable model, administering CCS with/without TCZ for CRS/ICANS management did not independently influence PFS (HR, 0.74; 95% CI, 0.36–1.51). These findings suggest that, among patients with relapsed/refractory MM, the timely and appropriate use of CCS or TCZ for mitigating immune-mediated toxicities does not appear to impact the antitumor activity and long-term outcomes of CAR T-cell therapy.

## Introduction

In recent years, autologous chimeric antigen receptor (CAR) T-cell therapy targeting B-cell maturation antigen (BCMA) has risen as an important treatment option for relapsed/refractory (R/R) multiple myeloma (MM) [1, 2]. Based on results from the phase 2 KarMMA trial, idecabtagene vicleucel (ide-cel) received US Food and Drug Administration approval for patients with at least four prior lines of therapy, including an immunomodulatory drug, a proteasome inhibitor, and an anti-CD38 monoclonal antibody (mAb) [3, 4]. Based on results from the phase 1b/2 CARTITUDE-1 trial, ciltacabtagene autoleucel (cilta-cel) was subsequently approved for the same indication [4, 5]. More recently, G protein-coupled receptor, class C, group 5, member D (GPRC5D) emerged as another actionable target in MM, with early-phase trials of GPRC5D-directed CAR T-cell products reporting promising outcomes [6–8].

Despite enabling remarkable responses, CAR T-cell therapy carries many inherent challenges that preempt its extended application in MM practice. For instance, a distinct set of adverse events can ensue, including cytokine release syndrome (CRS) and immune effector cell-associated neurotoxicity syndrome (ICANS) [9]. These immune-mediated toxicities are typically diagnosed within the first 4 weeks post-infusion, underscoring a critical period during which timely interventions may be required to mitigate their clinical repercussions [10, 11].

Tocilizumab (TCZ), a recombinant humanized mAb against the interleukin (IL)-6 receptor, has been proven effective in controlling the supraphysiologic inflammatory response that drives CRS [12]. In turn, corticosteroids (CCS) may also be incorporated in cases of severe, refractory, or protracted CRS events [11]. Owing to their ability to cross the blood-brain barrier, drugs like dexamethasone and methylprednisolone are also useful for ICANS management, with higher doses and/or prolonged courses sometimes being required in this setting [13].

Given their underlying mechanisms of action, immunosuppressants have the potential to interfere with the physiological activation, expansion, proliferation, survival, and function of circulating T cells [14–16]. This poses a theoretical risk to the optimal antitumor activity of CAR T-cell products, raising practical discussions on the adequate balance between mitigating treatment-related toxicities and preserving in vivo expansion/persistence, therapeutic efficacy, and clinical response [17, 18]. In view of the scarcity of MM-focused literature addressing this matter, we performed a retrospective study evaluating whether CCS or TCZ administration early after CAR T-cell infusion can influence long-term outcomes in patients with R/R MM.

## Methods

### Study design and population

This was a single-center, retrospective, observational cohort study of adult patients with R/R MM who underwent autologous CAR T-cell therapy targeting BCMA or GPRC5D (including commercial and investigational products) at the Memorial Sloan Kettering Cancer Center (MSKCC) between March 1, 2017, and February 28, 2023. The last follow-up date was August 1, 2023. Exclusion criteria encompassed prior CAR T-cell infusion at a different institution, enrollment in an ongoing clinical trial, and administration of an allogeneic product [19]. For subjects who received more than one CAR T-cell therapy at MSKCC over time, only the first product administered was considered in this analysis.

The research was conducted in accordance with the principles of the Declaration of Helsinki and received approval from the MSKCC Institutional Review Board (IRB). Due to its retrospective design, the requirement for patient-specific informed consent was waived by the IRB. For study reporting, Strengthening the Reporting of Observational Studies in Epidemiology (STROBE) guidelines for cohort studies were followed [20].

### Data collection and assessments

We undertook a retrospective collection of patient-level information, including demographics, baseline clinicopathologic characteristics, prior therapeutic interventions, CAR T-cell protocol details, and post-infusion clinical data. For individuals treated with a commercial product, data were sourced from electronic medical records (EMRs). For those treated with an investigational product, both EMRs and trial-specific case report forms (CRFs) were reviewed for pertinent information. A secured internal database, custom-designed with the Research Electronic Data Capture (REDCap) application, was employed for data storage. REDCap is a web-based software platform which provides an intuitive interface for validated data capture, audits trails for tracking data manipulation and export procedures, allows automated export procedures for seamless data downloads to common statistical packages, and facilitates procedures for data integration and interoperability with external sources [21].

Response assessments adhered to International Myeloma Working Group (IMWG) updated criteria, which define six categories: stringent complete response (sCR), complete response (CR), very good partial response (VGPR), partial response (PR), stable disease (SD), or progressive disease (PD) [22]. CRS and ICANS were graded according to American Society for Transplantation and Cellular Therapy guidelines [23]. Time parameters were calculated as the difference between the initial and final reference dates (e.g., if events occurred on a single calendar day, an interval of ‘0 days’ was recorded).

CCS and TCZ were prescribed at the discretion of the treating physicians and in adherence to the most recent MSKCC guidelines. To characterize individual exposure to each of these agents, we examined medication orders for both inpatient and outpatient prescriptions. This inspection covered the period up to 30 days following CAR T-cell infusion (timeframe during which immune-related adverse events typically occur) [24]. Total/cumulative doses of systemic CCS were quantified as dexamethasone equivalents (DexEqs; expressed in mg) [25].

### Study groups and outcome measures

To assess the influence of each examined intervention on efficacy outcomes, we stratified CAR T-cell-treated patients into two comparative cohorts. For the CCS analysis, subjects were allocated either into a CCS group (when exposed to systemic CCS within 30 days of CAR T-cell infusion) or a non-CCS group (when unexposed to systemic CCS within 30 days of CAR T-cell infusion). For the TCZ analysis, the study population was analogously divided into a TCZ group and a non-TCZ group.

The primary outcome was progression-free survival (PFS), which was calculated from the date of CAR T-cell infusion to the date of disease progression, last follow-up, or death from any cause (whichever occurred first). Secondary outcomes included the following measures: overall response rate (ORR; percentage of patients achieving PR or better), complete response rate (CRR; percentage of patients achieving CR or better), and overall survival (OS; time from date of CAR T-cell infusion to date of last follow-up or death from any cause).

### Statistical analysis

Descriptive statistics were reported as median (range) for continuous variables and number (percentage) for categorical variables. To compare response rates according to CCS/TCZ receipt, Pearson’s chi-squared (χ^2^) tests were employed. To compare survival outcomes according to CCS/TCZ receipt, we conducted a 30-day landmark analysis using Cox regression and Kaplan–Meier methodology. We also explored univariable Cox models to estimate the impact of other relevant factors on PFS and OS. To assess the independent impact of different covariates on the primary outcome measure, we evaluated a multivariable Cox regression model using the same 30-day landmark. Two-tailed tests were employed, and significance was defined at the 0.05 threshold for *p*-values. All statistical tests were done in R software version 4.2.2 (R Foundation for Statistical Computing, Vienna, Austria).

## Results

### Patients and treatment

Table 1 summarizes the baseline characteristics of the study population. Among the 101 patients included in the analysis (43% female), the median age was 62 years (range, 37–79) and median number of prior lines of therapy was 7 (range, 2–20). Notably, 83% had triple-class refractory disease, 49% had extramedullary disease, and 50% had high-risk cytogenetics. The specific rates of key cytogenetic abnormalities are detailed in Supplementary Table 1. While 5% had prior exposure to a bispecific T-cell engager, 15% had prior exposure to a non-cellular BCMA-directed agent. During the period between T-cell collection and lymphodepletion, 75% of subjects received a bridging regimen for disease control/debulking. In total, 92 patients (91%) received anti-BCMA CAR T cells and 9 patients (9%) received anti-GPRC5D CAR T cells.

**Table 1:** Baseline characteristics of the study population.

Characteristic	*N* = 101^a^
Median age (range) — yr	62 (37–79)
Median time from diagnosis (range) — yr	5.6 (1.0–23.6)
Sex — no. (%)	
Female	43 (43%)
Male	58 (57%)
Race — no. (%)	
White	76 (75%)
Black	15 (15%)
Other/Unknown	10 (10%)
Performance status — no. (%)	
ECOG 0	36 (36%)
ECOG 1	59 (58%)
ECOG 2	6 (6%)
Multiple myeloma subtype — no. (%)	
IgG	61 (61%)
IgA	22 (22%)
LC only	14 (14%)
Other/Unknown	4 (4%)
Predominant LC isotype — no. (%)	
Kappa LCs	57 (56%)
Lambda LCs	44 (44%)
High-risk cytogenetics^b^ — no. (%)	51 (50%)
Extramedullary disease — no. (%)	49 (49%)
High tumor burden^c^ — no. (%)	31 (31%)
Triple-class refractory^d^ — no. (%)	84 (83%)
Median number of prior therapy lines (range) — no.	7 (2–20)
Objective response to last therapy line — no. (%)	
Yes (PR or better)	55 (54%)
No (SD/PD only)	46 (46%)
Prior HDT/ASCT — no. (%)	98 (97%)
Prior BCMA-directed therapy^e^ — no. (%)	15 (15%)
Prior T-cell-redirecting therapy^f^ — no. (%)	5 (5%)
Bridging therapy^g^ — no. (%)	75 (75%)
Objective response to bridging regimen — no. (%)	
Yes (PR or better)	21 (21%)
No (SD/PD only)	54 (53%)
No bridging received	26 (26%)
Antigen targeted by CAR T-cell therapy — no. (%)	
BCMA	92 (91%)
GPRC5D	9 (9%)
Classification of CAR T-cell product — no. (%)	
Investigational	54 (53%)
Commercial^h^	47 (47%)

*BCMA* B-cell maturation antigen, *BsAb* bispecific antibody, *ECOG* Eastern Cooperative Oncology Group, *HDT/ASCT* high-dose chemotherapy followed by autologous hematopoietic stem cell transplant, *Ig* immunoglobulin, *LC* light chain, *PD* progressive disease, *PR* partial response, *SD* stable disease.

^a^Data pertains to the timepoint immediately before CAR T-cell infusion.

^b^High-risk cytogenetics was characterized by the presence of at least one of the following cytogenetic abnormalities: *t*(4;14), *t*(14;16), and/or del(17p).

^c^High tumor burden was characterized by the presence of ≥50% CD138-positive plasma cells in the last bone marrow biopsy performed before CAR T-cell infusion.

^d^Triple-class refractory disease was characterized by failure to achieve at least a PR or disease progression within 60 days after the last dose of an immunomodulatory agent, a proteasome inhibitor, and an anti-CD38 monoclonal antibody.

^e^Included either BCMA-directed BsAbs (e.g., teclistamab and elranatamab) or antibody-drug conjugates (e.g., belantamab mafodotin).

^f^Included BsAbs targeting BCMA (e.g., teclistamab and elranatamab), GPRC5D (e.g., talquetamab), or FcRH5 (e.g., cevostamab).

^g^Bridging therapy was defined as any anti-myeloma regimen given after leukapheresis and before lymphodepleting chemotherapy (e.g., low-dose fludarabine and cyclophosphamide) to achieve disease control/debulking while waiting for CAR T-cell manufacturing.

^h^Corresponds to all patients treated with commercially available products (including 7% of patients who received therapeutic doses of idecabtagene vicleucel or ciltacabtagene autoleucel as part of late-phase clinical trials).

### Efficacy and safety outcomes

At the data cut-off of August 2023, the median duration of follow-up was 27.4 months (interquartile range, 10.26–51.15 months). Following the administration of autologous CAR T-cell therapy, 76/101 patients (75%) developed CRS (34% grade 1; 34% grade 2; 8% grade 3) and 14/101 patients (14%) developed ICANS (8% grade 1; 4% grade 2; 2% grade 3). Table 2 provides an overview of CRS/ICANS events and their management.

**Table 2:** Overview of treatment protocol and associated clinical course.

Characteristic	*N* = 101
CRS events — no. (%)	
Any grade	76 (75%)
Grade 1	34 (34%)
Grade 2	34 (34%)
Grade ≥3	8 (8%)
Median time to CRS onset (range) — d	1 (0–14)
Median duration of CRS (range) — d	3 (0–10)
ICANS events — no. (%)	
Any grade	14 (14%)
Grade 1	8 (8%)
Grade 2	4 (4%)
Grade ≥3	2 (2%)
Median time to ICANS onset (range) — d	3.5 (0–29)
Median duration of ICANS (range) — d	5 (0–35)
TCZ use within 30 days post-infusion — no. (%)	49 (49%)
Median time to first TCZ dose (range) — d	1 (0–18)
Median duration of TCZ course (range) — d	0 (0–2)
CCS use within 30 days post-infusion — no. (%)	34 (34%)
Median time to first CCS dose (range) — d	2 (0–41)
Median duration of CCS course (range) — d	1 (0–52)
Median cumulative DexEq dose (range)^a^ — mg	20 (10–580)
Use of other immunosuppressants — no. (%)	
Siltuximab^b^	2 (2%)
Anakinra^c^	5 (5%)

*CCS* corticosteroid, *CRS* cytokine release syndrome, *DexEq* dexamethasone-equivalent, *ICANS* immune effector cell-associated neurotoxicity syndrome, *TCZ* tocilizumab.

^a^Calculated among patients who received CCS within 30 days of CAR T-cell infusion (*n* = 34).

^b^Anti-interleukin-6 chimeric monoclonal antibody.

^c^Recombinant human interleukin-1 receptor antagonist.

During the first month following CAR T-cell infusion, 49/101 patients (49%) received TCZ and 34/101 patients (34%) received systemic CCS, with a median cumulative DexEq dose of 20 mg for the latter group. Efficacy outcomes and survival curves for the whole cohort are displayed in Supplementary Table 2 and Supplementary Fig. 1, respectively.

### Corticosteroids analysis

**Fig. 1:**
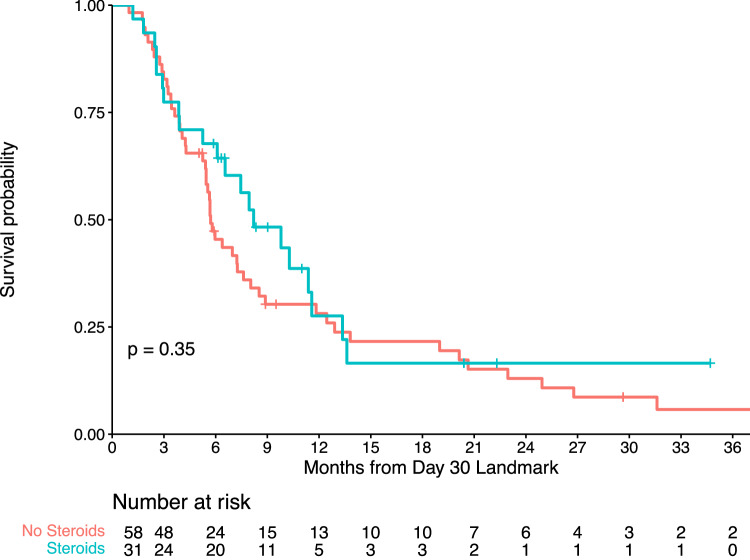
Steroid impact on progression-free survival. Kaplan-Meier plot comparing progression-free survival between patients exposed (blue curve) and unexposed (red curve) to corticosteroids within 30 days post-administration of CAR T-cell therapy for relapsed/refractory multiple myeloma.

In the Kaplan–Meier curve analysis, patients who received any dose of systemic CCS within 30 days of CAR T-cell infusion had similar PFS compared to those unexposed to CCS (Fig. 1). Correspondingly, Cox regression revealed no significant difference in PFS (HR, 0.78; 95% CI, 0.47–1.31; *p* = 0.34; Supplementary Table 3) or OS (HR, 1.77; 95% CI, 0.93–3.37; *p* = 0.09; Supplementary Table 4) when comparing these groups. As detailed in Table 3, CCS receipt also lacked a statistically significant impact on either ORR (85% versus 70%; *p* = 0.10) or CRR (38% versus 25%; *p* = 0.18).

**Table 3:** Response to CAR T-cell therapy based on exposure to corticosteroids or tocilizumab within 30 days post-infusion.

Response to CAR T cells	Non-TCZ group (*N* = 52)	TCZ group (*N* = 49)	Non-CCS group (*N* = 67)	CCS group (*N* = 34)
PR or better	35 (67%)	41 (84%)	47 (70%)	29 (85%)
No response	17 (33%)	8 (16%)	20 (30%)	5 (15%)
*p*-value	0.06		0.10	
CR/sCR	14 (27%)	16 (33%)	17 (25%)	13 (38%)
No CR/sCR	38 (73%)	33 (67%)	50 (75%)	21 (62%)
*p*-value	0.53		0.18	

*CAR* chimeric antigen receptor, *CR* complete response, *CCS* corticosteroids, *sCR* stringent complete response, *TCZ* tocilizumab.

Following stratification based on the median cumulative dose cut-off (20 mg in DexEqs), Kaplan–Meier curve analysis (Supplementary Fig. 2) and Cox regression (Supplementary Table 3) consistently showed a comparable PFS between >20 mg and 0–20 mg dose subgroups (HR, 0.94; 95% CI, 0.49–1.79; *p* = 0.84). No statistically significant association between total steroid dosing and OS was observed (HR, 2.05; 95% CI, 1.0–4.20; *p* = 0.07; Supplementary Table 4).

### Tocilizumab analysis

**Fig. 2:**
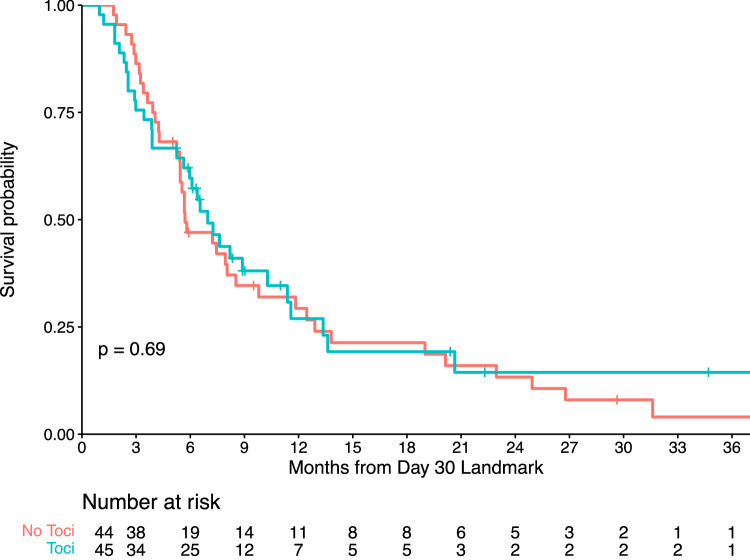
Tocilizumab impact on progression-free survival. Kaplan-Meier plot comparing progression-free survival between patients exposed (blue curve) and unexposed (red curve) to tocilizumab (Toci) within 30 days post-administration of CAR T-cell therapy for relapsed/refractory multiple myeloma.

In the Kaplan–Meier curve analysis, patients who received TCZ within 30 days of CAR T-cell infusion had similar PFS compared to those unexposed to TCZ (Fig. 2). Correspondingly, Cox regression revealed no significant difference in PFS (HR, 0.91; 95% CI, 0.57–1.45; *p* = 0.69; Supplementary Table 3) or OS (HR, 1.42; 95% CI, 0.76–2.66; *p* = 0.27; Supplementary Table 4) when comparing TCZ and non-TCZ groups. Furthermore, there was no significant difference in either ORR (84% versus 67%; *p* = 0.06) or CRR (33% versus 27%; *p* = 0.53) between these groups (Table 3).

### Multivariable model

From the univariable model for PFS (Supplementary Table 3), six clinically relevant covariates were selected for incorporation into a multivariable model: Eastern Cooperative Oncology Group Performance Status (ECOG) performance status, prior BCMA-directed therapy, high-risk cytogenetics, high tumor burden, bridging therapy, and CRS/ICANS management. The latter was included in the analysis as a four-strata combined variable: (1) No CRS/ICANS; (2) CRS/ICANS (no treatment); (3) CRS/ICANS (TCZ only); and (4) CRS/ICANS (CCS with/without TCZ). This was due to the expectedly strong association between CRS/ICANS occurrence and CCS/TCZ use.

After adjustment with multivariable Cox proportional hazards regression, poor performance status was found to be the only covariate independently associated with PFS (Table 4). Using ECOG 0 as the reference category, ECOG 1–2 was associated with an HR of 1.84 (95% CI, 1.09–3.12; *p* = 0.004). The presence of ≥50% bone marrow plasma cells in the pre-infusion biopsy was associated with a HR of 1.85, though statistical significance was not reached (95% CI, 0.98–3.52; *p* = 0.062). Employing a reference group of patients with CRS/ICANS who did not receive any immunosuppressants, the administration of CCS (either with or without TCZ) for CRS/ICANS management was found to lack an independent impact on PFS (HR, 0.74; 95% CI, 0.36–1.51; *p* = 0.30).

**Table 4:** Multivariable model assessing the independent impact of clinically relevant covariates on progression-free survival after CAR T-cell therapy for relapsed/refractory multiple myeloma.

Characteristic	*N*	Event *N*	HR	95% CI	*p*-value
Performance Status	86	69			0.021
ECOG 0			—	—	
ECOG 1–2			1.84	1.09, 3.12	
High-risk Cytogenetics	86	69			0.18
No			—	—	
Yes			1.44	0.84, 2.47	
Prior BCMA-directed Therapy	86	69			0.18
No			—	—	
Yes			1.78	0.78, 4.05	
Bridging Therapy	86	69			0.25
No			—	—	
Yes			1.45	0.76, 2.76	
High Tumor Burden	86	69			0.062
No (<50% BMPCs)			—	—	
Yes (≥50% BMPCs)			1.85	0.98, 3.52	
CRS/ICANS management	86	69			0.30
CRS/ICANS (No Treatment)			—	—	
No CRS/ICANS			1.56	0.75, 3.21	
CRS/ICANS (CCS ± TCZ)			0.74	0.36, 1.51	
CRS/ICANS (TCZ only)			1.11	0.50, 2.46	

*BMPC* bone marrow plasma cell, *BCMA* B-cell maturation antigen, *CCS* corticosteroid, *CI* confidence interval, *CRS* cytokine release syndrome, *ECOG* Eastern Cooperative Oncology Group, *HR* hazard ratio, *ICANS* immune effector cell-associated neurotoxicity syndrome, *TCZ* tocilizumab.

## Discussion

In this single-center, retrospective cohort study involving 101 patients who received investigational or commercial CAR T-cell therapy for R/R MM, we found no significant difference in PFS when comparing individuals exposed to CCS within 30 days post-infusion to those who were unexposed. ORR, CRR, and OS were also comparable between CCS and non-CCS groups. These findings remained consistent even after stratification based on cumulative DexEq dosing (≤20 mg versus >20 mg). Similarly, TCZ exposure within the first month following administration of anti-BCMA or anti-GPRC5D CAR T cells did not affect any of the efficacy endpoints analyzed.

While CCS are known to dampen T-cell activation signaling pathways and promote T-cell apoptosis, IL-6 blockade might hinder the post-stimulation cytokine response and expansion of effector T cells [15, 16, 26, 27]. Hence, researchers have questioned whether immunosuppressants can lead to a negative impact on CAR T-cell efficacy [28]. For instance, in a phase 1 trial of 19–28z CAR T cells for R/R B-cell acute lymphoblastic leukemia (B-ALL), Davila et al. suggested that steroid-induced lymphotoxicity contributed to some early relapses. Accordingly, participants exposed to high-dose CCS for severe CRS showed a 5-fold decrease in their bone marrow CAR T-cell counts when compared to those managed conservatively or with TCZ alone [29, 30]. Subsequent studies of CAR T-cell therapy for R/R B-ALL reached divergent conclusions. In a phase 1/2 study of SCRI-CAR19v1, neither TCZ nor CCS seemed to affect CAR T-cell expansion and persistence [31]. In a pooled analysis of three clinical trials investigating CD19- or CD22-targeted CAR T cells, the steroid group (*n* = 42) and non-steroid group (*n* = 26) demonstrated similar rates of minimal residual disease-negative CR (*p* = 0.249). Unexpectedly, average CAR T-cell numbers in peripheral blood on days 11, 15, 20, and 3 were significantly higher for the steroid group, suggesting that CCS may not suppress CAR T-cell proliferation [32].

R/R B-cell non-Hodgkin lymphoma (B-NHL) cohorts have also yielded conflicting evidence [28]. In a single-center analysis including 100 patients who received standard-of-care axicabtagene ciloleucel (axi-cel) for large B-cell lymphoma, higher cumulative doses of CCS (≥195 mg in DexEq) were associated with a lower PFS (*p* = 0.005). Moreover, a poorer OS was observed among participants exposed to CCS at any dose (*p* = 0.006), at higher cumulative doses (*p* < 0.001), within 7 days of CAR T-cell infusion (*p* = 0.005), or for a period ≥10 days (*p* = 0.003) [33]. Conversely, in a multicentric cohort of 298 axi-cel-treated individuals, neither TCZ use (HR, 1.7; 95% CI, 0.9–2.4; *p* = 0.17) nor CCS (HR, 1.3; 95% CI, 0.8–2.2; *p* = 0.2) were found to affect OS following a multivariable analysis using the day 30 landmark [34]. In parallel, two European cohorts and a recent US cohort showed no negative impact from these interventions on CAR T-cell efficacy for R/R B-NHL [35–37].

In the context of CAR T-cell therapy for R/R MM, the prognostic implications of immunosuppressant usage remains relatively underexplored. A retrospective study conducted in China involving 71 patients treated with anti-BCMA CAR T cells ± anti-CD19 CAR T cells found no significant differences in PFS (*p* = 0.22), OS (*p* = 0.47), ORR (*p* = 1.00), or CRR (*p* = 1.00) between the CCS and non-CCS groups. PFS was also unaffected by steroid dosing (*p* = 0.51 for >35 mg versus ≤35 mg in DexEqs), timing (*p* = 0.28 for ≤7-day versus >7-day timepoint), or duration (*p* = 0.41 for ≤3 days versus >3 days) [38]. In a single-center cohort of US patients treated with anti-BCMA CAR T cells, those given 0, ≤60, or >60 mg cumulative steroid dose had comparable PFS (*p* = 0.50), OS (*p* = 0.58), and time to next treatment (TTNT; *p* = 0.65). For patients who received 0, 1–5, or ≥5 days of CCS, a significantly shorter TTNT was noted for the latter subgroup (*p* = 0.04), though no significant differences in PFS and OS were observed [39].

Both the above studies, limited by small sample sizes, lacked a multivariable model accounting for additional factors that could impact outcomes. To the best of our knowledge, our retrospective analysis represents the largest examination to date of the influence of CCS and TCZ on CAR T-cell efficacy for R/R MM. Notably, the distribution of patients receiving CCS (34%), TCZ (49%), or anakinra (5%) in this cohort align with the percentages reported in a recent real-world experience on standard-of-care ide-cel (26%, 71%, and 5%, respectively) [40]. The present study is also notable for being the first to encompass patients treated with both anti-BCMA and anti-GPRC5D therapies. Furthermore, we employed a comprehensive multivariable model to adjust for key variables potentially influencing efficacy endpoints. Our results affirm the strategic and judicious use of TCZ and CCS for treating immune-mediated toxicities in patients receiving CAR T-cell therapy for MM. Such insights gain additional significance as initiatives are in progress to expand the application of CAR T-cell therapy to earlier stages of MM treatment and across more diverse healthcare settings, underscoring the need for refined supportive strategies in broader clinical contexts [41–43].

However, a few study limitations merit consideration. First, the monocentric and retrospective nature of this analysis inherently carries potential biases; therefore, caution should be exercised when extrapolating our results to broader patient populations or different clinical settings. Second, the variability in the CAR T-cell products administered presents a challenge in drawing specific conclusions about individual products. Herewith, the relatively low number of patients who received an anti-GPRC5D product limited our ability to perform a robust subgroup analysis. Furthermore, the present report lacked data on CAR T-cell dynamics/kinetics by peripheral blood analysis, which should be considered in future research endeavors [44]. Although the median PFS observed (6.5 months) was lower when compared to prior reports, 53% of patients received an investigational product as part of a clinical trial (including dose-escalation trials) and 15% had been previously exposed to a BCMA-directed therapy, which was an exclusion criterion in both KarMMA and CARTITUDE-1. Besides not having established a minimum follow-up period for patient inclusion in survival analyses, our follow-up period was considerably longer for patients receiving investigational products, with the first CAR T-cell infusion dating back to March 2017 (nearly five years prior to cilta-cel approval and four years prior to ide-cel approval).

In conclusion, our retrospective cohort study suggests that neither TCZ nor CCS use compromises the antitumor efficacy and long-term outcomes of CAR T-cell therapy for R/R MM, supporting the timely and appropriate administration of these immunosuppressants to attenuate CRS or ICANS events. Future studies expanding to larger patient populations and employing advanced diagnostics (e.g., circulating CAR T-cell assays) will be instrumental to further refine management strategies for immune-mediated toxicities while upholding the promise of CAR T-cell therapy in modern-day MM care.

### Supplementary information

Supplementary Material

## Data Availability

Individual participant data will not be shared because patients have not consented to share data with third parties.

## References

[1] Mikkilineni L, Kochenderfer JN (2021). CAR T cell therapies for patients with multiple myeloma. Nat Rev Clin Oncol.

[2] Banerjee R, Lee SS, Cowan AJ (2022). Innovation in BCMA CAR-T therapy: building beyond the Model T. Front Oncol.

[3] Munshi NC, Anderson LD, Shah N, Madduri D, Berdeja J, Lonial S (2021). Idecabtagene vicleucel in relapsed and refractory multiple myeloma. N Engl J Med.

[4] Holstein SA, Grant SJ, Wildes TM (2023). Chimeric antigen receptor T-cell and bispecific antibody therapy in multiple myeloma: moving into the future. J Clin Oncol.

[5] Berdeja JG, Madduri D, Usmani SZ, Jakubowiak A, Agha M, Cohen AD (2021). Ciltacabtagene autoleucel, a B-cell maturation antigen-directed chimeric antigen receptor T-cell therapy in patients with relapsed or refractory multiple myeloma (CARTITUDE-1): a phase 1b/2 open-label study. Lancet.

[6] Mailankody S, Devlin SM, Landa J, Nath K, Diamonte C, Carstens EJ (2022). GPRC5D-targeted CAR T cells for myeloma. N Engl J Med.

[7] Zhang M, Wei G, Zhou L, Zhou J, Chen S, Zhang W (2023). GPRC5D CAR T cells (OriCAR-017) in patients with relapsed or refractory multiple myeloma (POLARIS): a first-in-human, single-centre, single-arm, phase 1 trial. Lancet Haematol.

[8] Xia J, Li H, Yan Z, Zhou D, Wang Y, Qi Y (2023). Anti–G protein–coupled receptor, class C group 5 member D chimeric antigen receptor T cells in patients with relapsed or refractory multiple myeloma: a single-arm, phase II trial. J Clin Oncol.

[9] Santomasso B, Bachier C, Westin J, Rezvani K, Shpall EJ (2019). The other side of CAR T-cell therapy: cytokine release syndrome, neurologic toxicity, and financial burden. Am Soc Clin Oncol Educ Book.

[10] Morris EC, Neelapu SS, Giavridis T, Sadelain M (2022). Cytokine release syndrome and associated neurotoxicity in cancer immunotherapy. Nat Rev Immunol.

[11] Santomasso BD, Nastoupil LJ, Adkins S, Lacchetti C, Schneider BJ, Anadkat M (2021). Management of immune-related adverse events in patients treated with chimeric antigen receptor T-cell therapy: ASCO guideline. J Clin Oncol.

[12] Banerjee R, Marsal J, Huang CY, Lo M, Kambhampati S, Kennedy VE (2021). Early time-to-tocilizumab after B cell maturation antigen-directed chimeric antigen receptor T cell therapy in myeloma. Transpl Cell Ther.

[13] Jain MD, Smith M, Shah NN. How I treat refractory CRS and ICANS following CAR T-cell therapy. Blood. 2023. 10.1182/blood.2022017414 10.1182/blood.2022017414 PMC10329191 36989488

[14] Strauss G, Osen W, Debatin KM (2002). Induction of apoptosis and modulation of activation and effector function in T cells by immunosuppressive drugs. Clin Exp Immunol.

[15] Liberman AC, Budziñski ML, Sokn C, Gobbini RP, Steininger A, Arzt E (2018). Regulatory and mechanistic actions of glucocorticoids on T and inflammatory cells. Front Endocrinol.

[16] Chandran S, Leung J, Hu C, Laszik ZG, Tang Q, Vincenti FG (2021). Interleukin-6 blockade with tocilizumab increases Tregs and reduces T effector cytokines in renal graft inflammation: a randomized controlled trial. Am J Transpl.

[17] Hashmi H, McGann M, Greenwell BI (2023). Use of long-term corticosteroids in patients treated with CAR T-cell therapy. J Oncol Pharm Pr.

[18] Dickinson MJ, Barba P, Jäger U, Shah NN, Blaise D, Briones J (2023). A novel autologous CAR-T therapy, YTB323, with preserved T-cell stemness shows enhanced CAR T-cell efficacy in preclinical and early clinical development. Cancer Discov.

[19] Mailankody S, Matous JV, Chhabra S, Liedtke M, Sidana S, Oluwole OO (2023). Allogeneic BCMA-targeting CAR T cells in relapsed/refractory multiple myeloma: phase 1 UNIVERSAL trial interim results. Nat Med.

[20] Von Elm E, Altman DG, Egger M, Pocock SJ, Gøtzsche PC, Vandenbroucke JP (2007). The strengthening the reporting of observational studies in epidemiology (STROBE) statement: guidelines for reporting observational studies. Lancet.

[21] Harris PA, Taylor R, Minor BL, Elliott V, Fernandez M, O’Neal L (2019). The REDCap consortium: building an international community of software partners. J Biomed Inf.

[22] Kumar S, Paiva B, Anderson KC, Durie B, Landgren O, Moreau P (2016). International Myeloma Working Group consensus criteria for response and minimal residual disease assessment in multiple myeloma. Lancet Oncol.

[23] Lee DW, Santomasso BD, Locke FL, Ghobadi A, Turtle CJ, Brudno JN (2019). ASTCT consensus grading for cytokine release syndrome and neurologic toxicity associated with immune effector cells. Biol Blood Marrow Transpl.

[24] Liang EC, Sidana S (2023). Managing side effects: guidance for use of immunotherapies in multiple myeloma. Hematology.

[25] Liu D, Ahmet A, Ward L, Krishnamoorthy P, Mandelcorn ED, Leigh R (2013). A practical guide to the monitoring and management of the complications of systemic corticosteroid therapy. Allergy Asthma Clin Immunol.

[26] Herold MJ, McPherson KG, Reichardt HM (2006). Glucocorticoids in T cell apoptosis and function. Cell Mol Life Sci.

[27] Beyranvand Nejad E, Labrie C, Van Elsas MJ, Kleinovink JW, Mittrücker HW, Franken KLMC (2021). IL-6 signaling in macrophages is required for immunotherapy-driven regression of tumors. J Immunother Cancer.

[28] Sun Z, Xun R, Liu M, Wu X, Qu H (2021). The association between glucocorticoid administration and the risk of impaired efficacy of axicabtagene ciloleucel treatment: a systematic review. Front Immunol.

[29] Davila ML, Riviere I, Wang X, Bartido S, Park J, Curran K, et al. Efficacy and toxicity management of 19-28z CAR T cell therapy in B cell acute lymphoblastic leukemia. *Sci Transl Med*. 2014;6. 10.1126/scitranslmed.3008226 10.1126/scitranslmed.3008226 PMC4684949 24553386

[30] Brentjens RJ, Davila ML, Riviere I, Park J, Wang X, Cowell LG, et al. CD19-targeted T cells rapidly induce molecular remissions in adults with chemotherapy-refractory acute lymphoblastic leukemia. *Sci Transl Med*. 2013;5. 10.1126/scitranslmed.3005930 10.1126/scitranslmed.3005930 PMC3742551 23515080

[31] Gardner RA, Ceppi F, Rivers J, Annesley C, Summers C, Taraseviciute A (2019). Preemptive mitigation of CD19 CAR T-cell cytokine release syndrome without attenuation of antileukemic efficacy. Blood.

[32] Liu S, Deng B, Yin Z, Pan J, Lin Y, Ling Z (2020). Corticosteroids do not influence the efficacy and kinetics of CAR-T cells for B-cell acute lymphoblastic leukemia. Blood Cancer J.

[33] Strati P, Ahmed S, Furqan F, Fayad LE, Lee HJ, Iyer SP (2021). Prognostic impact of corticosteroids on efficacy of chimeric antigen receptor T-cell therapy in large B-cell lymphoma. Blood.

[34] Nastoupil LJ, Jain MD, Feng L, Spiegel JY, Ghobadi A, Lin Y (2020). Standard-of-care axicabtagene ciloleucel for relapsed or refractory large B-cell lymphoma: results from the US lymphoma CAR T consortium. J Clin Oncol.

[35] Sesques P, Ferrant E, Safar V, Wallet F, Tordo J, Dhomps A (2020). Commercial anti-CD19 CAR T cell therapy for patients with relapsed/refractory aggressive B cell lymphoma in a European center. Am J Hematol.

[36] Lakomy T, Akhoundova D, Nilius H, Kronig MN, Novak U, Daskalakis M (2023). Early use of corticosteroids following CAR T-cell therapy correlates with reduced risk of high-grade CRS without negative impact on neurotoxicity or treatment outcome. Biomolecules.

[37] Luttwak E, Flynn JR, Devlin SM, Cassanello G, Corona M, Dahi PB (2023). Patterns and safety of glucocorticosteroids use following CD19 CAR-T cell therapy for B-cell lymphoma. Blood.

[38] Wang X, Qi Y, Li H, Liu F, Cao J, Chen W (2022). Impact of glucocorticoids on short-term and long-term outcomes in patients with relapsed/refractory multiple myeloma treated with CAR-T therapy. Front Immunol.

[39] Duvalyan E, Shah N, Lo M, Martin T, Wolf JL, Chung A (2023). Impact of corticosteroids on efficacy of BCMA targeted CAR-T therapy in multiple myeloma. Leuk Lymphoma.

[40] Zhang X, Zhang H, Lan H, Wu J, Xiao Y (2023). CAR-T cell therapy in multiple myeloma: current limitations and potential strategies. Front Immunol.

[41] Hansen DK, Sidana S, Peres LC, Leitzinger CC, Shune L, Shrewsbury A (2023). Idecabtagene vicleucel for relapsed/refractory multiple myeloma: real-world experience from the myeloma CAR T consortium. J Clin Oncol.

[42] Shah P, Sperling AS (2023). Chimeric antigen receptor T cells in multiple myeloma. Hematol Oncol Clin North Am.

[43] Sadek NL, Costa BA, Nath K, Mailankody S (2023). CAR T‐cell therapy for multiple myeloma: a clinical practice‐oriented review. Clin Pharm Ther.

[44] Sarikonda G, Pahuja A, Kalfoglou C, Burns K, Nguyen K, Ch’en IL (2021). Monitoring CAR-T cell kinetics in clinical trials by multiparametric flow cytometry: benefits and challenges. Cytom B Clin Cytom.

