# Supplementary material for: Prognostic impact of corticosteroid and tocilizumab use following chimeric antigen receptor T-cell therapy for multiple myeloma
Source: Blood Cancer J. 2024 May 27;14(1):84. doi: 10.1038/s41408-024-01048-0 (PMC11130279; doi:10.1038/s41408-024-01048-0)

**SUPPLEMENTAL MATERIAL**

**Supplemental Table 1:** Incidence of key cytogenetic abnormalities among study participants.

| **Cytogenetic abnormalities — no. (%)** | **N=101** |
| --- | --- |
| 1q gain | 67 (66%) |
| 13q deletion | 54 (53%) |
| 17p deletion | 38 (38%) |
| 1p deletion | 23 (23%) |
| t(11;14) translocation | 19 (19%) |
| t(14;16) translocation | 8 (8%) |
| t(14;20) translocation | 2 (2%) |

**Supplemental Table 2:** Efficacy outcomes for the entire cohort.

| **Efficacy endpoints** | **N=101** |
| --- | --- |
| Best overall response* — no. (%) | |
| Stringent complete response (sCR) | 24 (24%) |
| Complete response (CR) | 6 (6%) |
| Very good partial response (VGPR) | 27 (27%) |
| Partial response (PR) | 19 (19%) |
| Stable disease (SD) | 14 (14%) |
| Progressive disease (PD) | 11 (11%) |
| Overall response rate (PR or better) | 75% |
| Complete response rate (CR or better) | 30% |
| Median PFS (95% CI) — mo. | 6.5 (5.6–8.5) |
| Median OS (95% CI) — mo. | 22 (15–54) |

*CI*, confidence interval; *OS*, overall survival; *PFS*, progression-free survival.

**Supplemental Table 3:** Univariable Cox proportional hazards model assessing the correlation between individual clinically-relevant covariates and progression-free survival (PFS).

| **Characteristic** | **N** | **Event N** | **HR** | **95% CI** | ***p*-value** |
| --- | --- | --- | --- | --- | --- |
| **Age at CAR T-cell infusion** | 89 | 71 | 1.00 | 0.98, 1.02 | 0.82 |
| **Sex** | 89 | 71 |  |  | 0.70 |
| Female |  |  | — | — |  |
| Male |  |  | 1.10 | 0.68, 1.76 |  |
| **Performance Status** | 89 | 71 |  |  | **0.007** |
| ECOG 0 |  |  | — | — |  |
| ECOG 1-2 |  |  | 1.94 | 1.19, 3.16 |  |
| **High-risk Cytogenetics** | 89 | 71 |  |  | 0.056 |
| No |  |  | — | — |  |
| Yes |  |  | 1.63 | 0.99, 2.69 |  |
| **High Tumor Burden** | 87 | 69 |  |  | 0.24 |
| No (<50% BMPCs) |  |  | — | — |  |
| Yes (≥50% BMPCs) |  |  | 1.36 | 0.82, 2.28 |  |
| **Number of Prior Therapy Lines** | 89 | 71 | 0.97 | 0.89, 1.05 | 0.47 |
| **Double Refractory** | 89 | 71 |  |  | 0.32 |
| No |  |  | — | — |  |
| Yes |  |  | 0.63 | 0.27, 1.47 |  |
| **Triple-class Refractory** | 89 | 71 |  |  | 0.55 |
| No |  |  | — | — |  |
| Yes |  |  | 0.82 | 0.44, 1.54 |  |
| **Prior ASCT** | 89 | 71 |  |  | 0.84 |
| No |  |  | — | — |  |
| Yes |  |  | 1.22 | 0.17, 8.90 |  |
| **Prior BCMA-directed Therapy** | 89 | 71 |  |  | 0.077 |
| No |  |  | — | — |  |
| Yes |  |  | 1.95 | 0.98, 3.91 |  |
| **Prior T-cell-redirecting Therapy** | 89 | 71 |  |  | 0.64 |
| No |  |  | — | — |  |
| Yes |  |  | 0.73 | 0.18, 2.98 |  |
| **Bridging Therapy** | 88 | 71 |  |  | 0.067 |
| No |  |  | — | — |  |
| Yes |  |  | 1.68 | 0.94, 3.00 |  |
| **CRS Developed** | 89 | 71 |  |  | 0.18 |
| No |  |  | — | — |  |
| Yes |  |  | 0.66 | 0.37, 1.18 |  |
| **ICANS Developed** | 89 | 71 |  |  | 0.59 |
| No |  |  | — | — |  |
| Yes |  |  | 1.21 | 0.62, 2.33 |  |
| **TCZ within 30 Days of Infusion** | 89 | 71 | 0.91 | 0.57, 1.45 | 0.69 |
| **CCS within 30 Days of Infusion** | 89 | 71 | 0.78 | 0.47, 1.31 | 0.34 |
| **Cumulative DexEq Dose** | 89 | 71 |  |  | 0.84 |
| No CCS or median dose ≤20 mg |  |  | — | — |  |
| Median dose >20 mg |  |  | 0.94 | 0.49, 1.79 |  |
| **Response to Last Therapy Line** | 89 | 71 |  |  | 0.50 |
| PR or Better |  |  | — | — |  |
| SD/PD |  |  | 0.85 | 0.53, 1.36 |  |
| **Response to Bridging Therapy** | 65 | 54 |  |  | 0.43 |
| PR or Better |  |  | — | — |  |
| SD/PD |  |  | 0.79 | 0.44, 1.42 |  |
| **CRS/ICANS management** | 89 | 71 |  |  | 0.40 |
| CRS/ICANS (No Treatment) |  |  | — | — |  |
| No CRS/ICANS |  |  | 1.61 | 0.82, 3.15 |  |
| CRS/ICANS (CCS ± TCZ) |  |  | 0.97 | 0.52, 1.79 |  |
| CRS/ICANS (TCZ only) |  |  | 1.38 | 0.69, 2.72 |  |
| Abbreviations: *ASCT*, autologous stem cell transplantation; *BMPC*, bone marrow plasma cell; *BCMA*, B-cell maturation antigen; *CCS*, corticosteroids; *CI*, confidence interval; *CRS*, cytokine release syndrome; *DexEq*, dexamethasone equivalent; Eastern Cooperative Oncology Group; *ECOG*, Eastern Cooperative Oncology Group; *HR*, hazard ratio; *ICANS*, immune effector cell-associated neurotoxicity syndrome; *PD*, progressive disease; *PR*, partial response; *SD*, stable disease; *TCZ*, tocilizumab. | | | | | |

**Supplemental Table 4:** Univariable Cox proportional hazards model assessing the correlation between individual clinically-relevant covariates and overall survival.

| **Characteristic** | **N** | **Event N** | **HR** | **95% CI** | ***p*-value** |
| --- | --- | --- | --- | --- | --- |
| **Age at CAR T-cell infusion** | 89 | 43 | 1.02 | 0.99, 1.05 | 0.26 |
| **Sex** | 89 | 43 |  |  | 0.39 |
| Female |  |  | — | — |  |
| Male |  |  | 0.77 | 0.42, 1.40 |  |
| **Performance Status** | 89 | 43 |  |  | **0.007** |
| ECOG 0 |  |  | — | — |  |
| ECOG 1-2 |  |  | 2.34 | 1.23, 4.45 |  |
| **High-risk Cytogenetics** | 89 | 43 |  |  | 0.39 |
| No |  |  | — | — |  |
| Yes |  |  | 1.30 | 0.71, 2.37 |  |
| **High Tumor Burden** | 87 | 42 |  |  | 0.25 |
| No (<50% BMPCs) |  |  | — | — |  |
| Yes (≥50% BMPCs) |  |  | 1.47 | 0.78, 2.77 |  |
| **Number of Prior Therapy Lines** | 89 | 43 | 0.99 | 0.89, 1.10 | 0.81 |
| **Double Refractory** | 89 | 43 |  |  | 0.079 |
| No |  |  | — | — |  |
| Yes |  |  | 0.34 | 0.12, 0.98 |  |
| **Triple-class Refractory** | 89 | 43 |  |  | **0.043** |
| No |  |  | — | — |  |
| Yes |  |  | 0.43 | 0.21, 0.91 |  |
| **Prior ASCT** | 89 | 43 |  |  | 0.50 |
| No |  |  | — | — |  |
| Yes |  |  | 0.46 | 0.06, 3.43 |  |
| **Prior BCMA-directed Therapy** | 89 | 43 |  |  | 0.87 |
| No |  |  | — | — |  |
| Yes |  |  | 1.10 | 0.39, 3.11 |  |
| **Prior T-cell-redirecting Therapy** | 89 | 43 |  |  | 0.054 |
| No |  |  | — | — |  |
| Yes |  |  | 0.00 | 0.00, Inf |  |
| **Bridging Therapy** | 88 | 43 |  |  | 0.22 |
| No |  |  | — | — |  |
| Yes |  |  | 1.58 | 0.73, 3.43 |  |
| **CRS Developed** | 89 | 43 |  |  | 0.24 |
| No |  |  | — | — |  |
| Yes |  |  | 1.71 | 0.66, 4.40 |  |
| **ICANS Developed** | 89 | 43 |  |  | **0.035** |
| No |  |  | — | — |  |
| Yes |  |  | 2.29 | 1.12, 4.67 |  |
| **TCZ within 30 Days of Infusion** | 89 | 43 | 1.42 | 0.76, 2.66 | 0.27 |
| **CCS within 30 Days of Infusion** | 89 | 43 | 1.77 | 0.93, 3.37 | 0.089 |
| **Cumulative DexEq Dose** | 89 | 43 |  |  | 0.066 |
| No CCS or median dose ≤20 mg |  |  | — | — |  |
| Median dose >20 mg |  |  | 2.05 | 1.00, 4.20 |  |
| **Response to Last Therapy Line** | 89 | 43 |  |  | 0.69 |
| PR or Better |  |  | — | — |  |
| SD/PD |  |  | 0.88 | 0.48, 1.64 |  |
| **Response to Bridging Therapy** | 65 | 35 |  |  | 0.60 |
| PR or Better |  |  | — | — |  |
| SD/PD |  |  | 0.82 | 0.39, 1.71 |  |
| **CRS/ICANS management** | 89 | 43 |  |  | 0.32 |
| CRS/ICANS (No Treatment) |  |  | — | — |  |
| No CRS/ICANS |  |  | 0.70 | 0.25, 1.94 |  |
| CRS/ICANS (CCS ± TCZ) |  |  | 1.67 | 0.80, 3.49 |  |
| CRS/ICANS (TCZ only) |  |  | 1.04 | 0.42, 2.60 |  |
| Abbreviations: *ASCT*, autologous stem cell transplantation; *BMPC*, bone marrow plasma cell; *BCMA*, B-cell maturation antigen; *CCS*, corticosteroids; *CI*, confidence interval; *CRS*, cytokine release syndrome; *DexEq*, dexamethasone equivalent; *ECOG*, Eastern Cooperative Oncology Group; *HR*, hazard ratio; *ICANS*, immune effector cell-associated neurotoxicity syndrome; *PD*, progressive disease; *PR*, partial response; *SD*, stable disease; *TCZ*, tocilizumab. | | | | | |

**Supplemental Figure 1:** Progression-free survival (A) and overall survival (B) curves for the entire cohort (N=101).


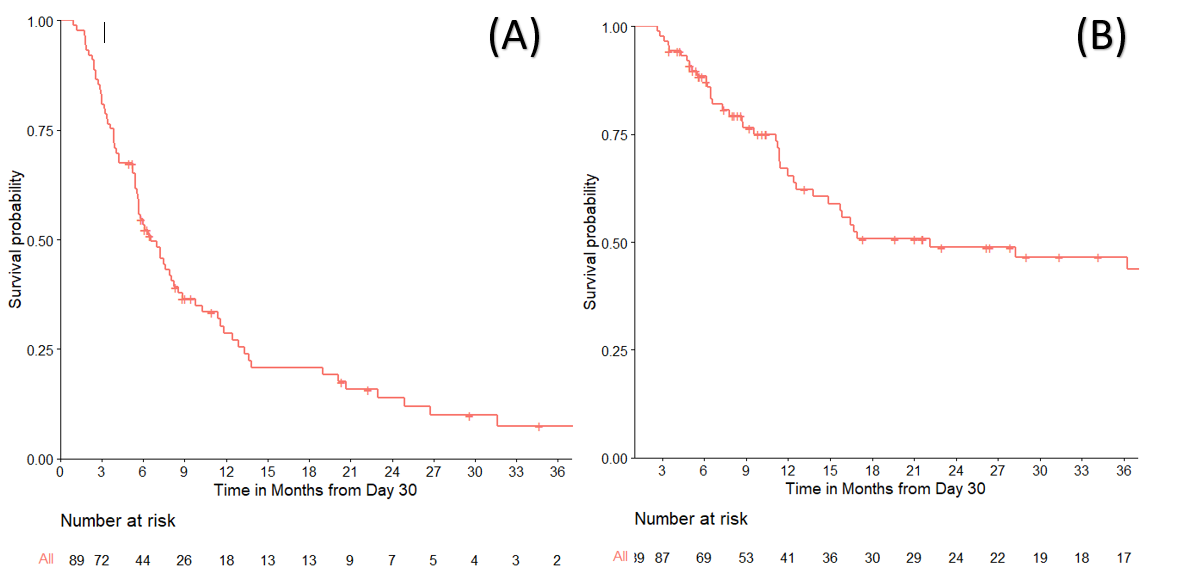


**Supplemental Figure 2:** Progression-free survival (PFS) impact of cumulative steroid doses within 30 days of CAR T-cell therapy for relapsed/refractory multiple myeloma.


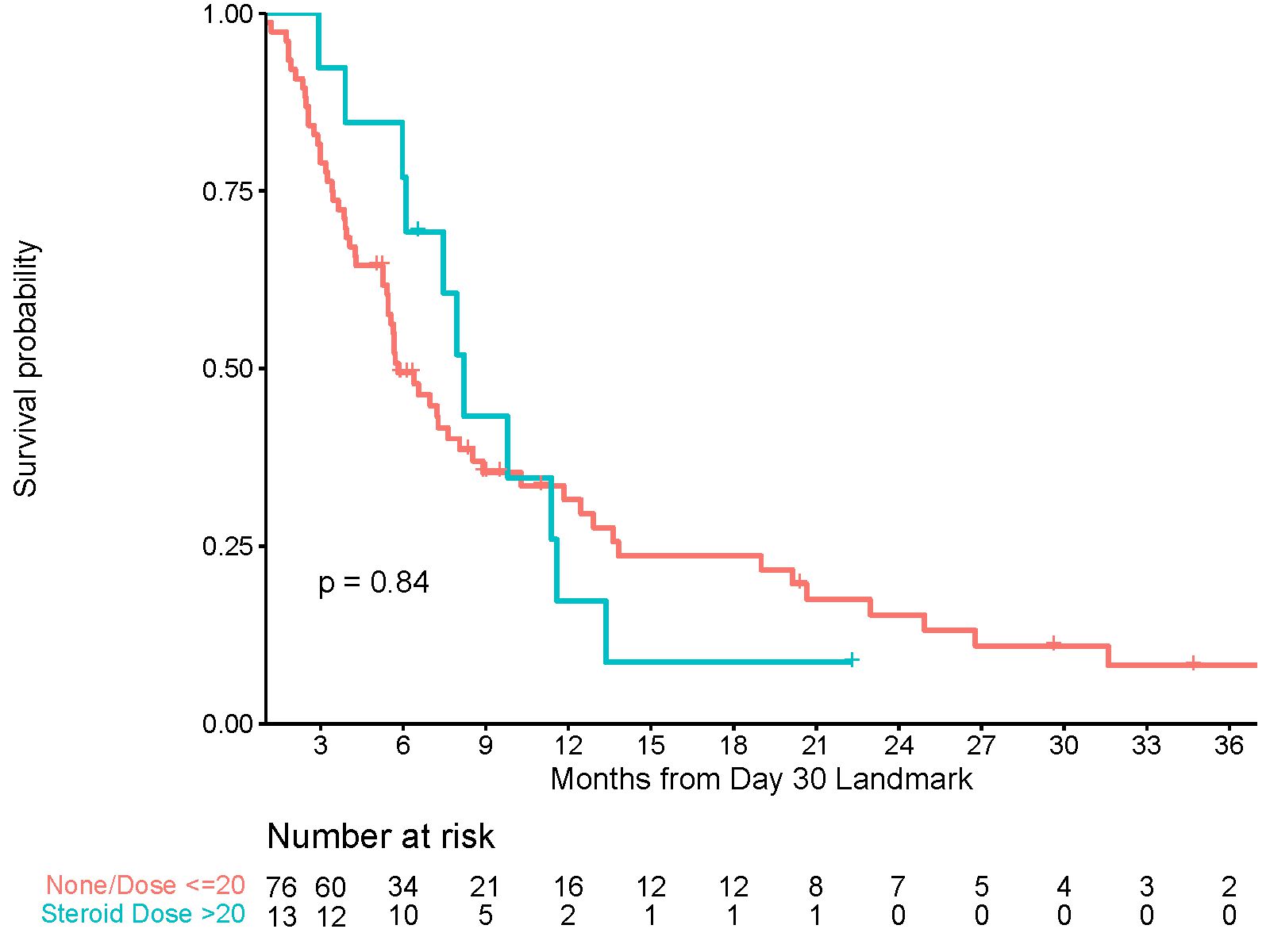

Supplement: Supplementary file 1 — Supplementary Material [file 41408_2024_1048_MOESM1_ESM.docx]
